# Medical education and the COVID-19 pandemic – a dress rehearsal for the “climate pandemic”?

**DOI:** 10.3205/zma001425

**Published:** 2021-01-28

**Authors:** Christoph Nikendei, Anna Cranz, Till Johannes Bugaj

**Affiliations:** 1 University Hospital Heidelberg, Department for General Internal Medicine and Psychosomatics, Heidelberg, Germany

**Keywords:** COVID-19, medical education, planetary health, climate change

## Abstract

In the present commentary, we raise the question whether the COVID-19 pandemic should be seen as just the dress rehearsal for what awaits us in the impending climate crisis. Many factors have helped us navigate the challenge of this coronavirus pandemic and continue to do so. These include: recognizing scientific expertise, medical education, and digitalization as important driving forces, providing us with key information about the SARS-CoV-2 virus, as well as integrating it into our curricula and promoting action-oriented research. However, the “premiere of the climate pandemic” will, in all likelihood, confront us with even greater challenges, difficulties, and threats. Adhering to scientific findings, promoting medical education about the effects of global warming and using the power of digitalization, as well as consciously engaging in our role as medical caregivers and leaders will make a decisive contribution to providing impetus for climate action.

## Zusammenfassung

Der vorliegende Kommentar wirft die Frage auf, ob die COVID-19-Pandemie als eine Generalprobe für das zu sehen ist, was uns in der bevorstehenden Klimakrise erwartet. Eine Vielzahl an Faktoren hat dazu beitragen, dass wir die Herausforderung der Corona-Pandemie aktiv bewältigen konnten und können. Hierzu gehören: die Integration von Wissenschaftlichkeit, der Einbezug von Medizinstudierenden, Lehre und Digitalisierung als Impulsgeber, Informationen über das SARS-CoV-2-Virus, deren Integration in die Curricula und nicht zuletzt eine handlungsbezogene Forschung. Die Klimakrise wird uns jedoch im Sinne einer „Premiere der Klima-Pandemie“ aller Voraussicht nach vor noch größere Bedrohungen und Schwierigkeiten stellen. Die Beachtung und Integration wissenschaftlicher Evidenz, die Lehre über die Auswirkungen der globalen Erwärmung, die bewusste Wahrnehmung ärztlicher Rollen- und Vorbildfunktionen sowie die Digitalisierung können dabei als Handlungsimpulse von besonderer Relevanz sein.

## SARS-CoV-2–catastrophe – a dress rehearsal

The curtain drops for the interlude of summer 2020. Solitary clapping in the auditorium. Well, it nearly worked. Still a lot of room for improvement, but it was all right for a dress rehearsal. Still some time before things get really serious. Thank goodness for that. There is still some time to think about those crucial issues – to reflect, focus, and revise key elements again. Unlike a theatre dress rehearsal, the SARS-CoV-2-catastrophe hit us suddenly and largely unforeseen impressing upon us concrete, increasingly graphic images of what global threat means. In many countries, it is thanks to the level-headed, reflected, and science-based approach of those working in health policy and the health sector – including medical students - that we were able to tread the boards for this dress rehearsal. Although the cost has high.

Alongside physicians, medical students bravely engaged in infection prevention, providing immediate assistance and care to patients suspected of being infected with SARS-CoV-2 or COVID-19 patients [1]. At the same time, telemedical approaches sored to the forefront of medical training and the support of patients – impacting future physicians directly [2], [3]. Online learning services [4] and, even in the field of simulation-based learning, distance learning models [5] were quickly introduced to medical curricula everywhere. The virus itself has become a focus of medical education. In no time at all, research funds were mobilized and scientists, together with their PhD students and research assistants, set out in search of effective treatment methods and a vaccine. Despite considerable challenges and psychological burden [6], medical students have shown their particular resilience: conviction regarding scientific data on reproduction rates, the effectiveness of preventive measures and therapeutic approaches, and trust in our technological resources may well have helped us cheat the gallows as much as possible and leave us very shaken but possibly better prepared for future SARS-CoV-2 outbreaks.

## It’s getting serious – the climate pandemic

It seems all the more cynical that this trust in feasibility, our experience of invulnerability, our fixation on technology and the continued expansion of “global reach” [7] will lead us directly towards the staging of the “climate pandemic”. Global warming appears to be less tangible, less comprehensible, even less daunting at times. Yet, sadly, this is a dangerous deception. Rising temperatures and sea levels will make large parts of the earth uninhabitable. By 2050, 140 million additional climate refugees will be on their way to less hostile regions, and as a result social structures and economic areas will begin to destabilize [8]. Contrary to the haphazardly staged COVID-19 dress rehearsal, we are able to plan and prepare our performance in the “climate pandemic”.

Over 26,000 scientists have publicly declared that the demands voiced by the Fridays-for-Future movement must be taken seriously and are based on an accurate interpretation of scientific data and findings [9]. The “Lancet Countdown on Health and Climate Change” [10] concludes that our society's and our children's health largely depends on how we deal with climate change. More than ever before, and despite all the already existing physician-led initiatives and efforts, we as (future) medical doctors need to ask ourselves the following questions:

- Why are we not making the scientific data on the current and imminent effects of global warming our guiding principle for action and treating the findings just as seriously as we are and have been with the COVID-19 pandemic [11]?
- Why are we only partially assuming our responsibility as role models and medical practitioners when it comes to global warming [12]?
- Why are we still insufficiently qualified with regard to the medical implications of climate change [13]?
- Why are we not ensuring that the places where we are trained and our patients are treated become mentally and physically sustainable environments and evolve into “green hospitals” [14]?
- Why don’t we use the “miracle cure” of digitalization, the technology that was successfully used to ensure distance learning and treatment approaches during the COVID-19 pandemic, to make an important contribution towards minimizing our climate impact in light of the impending “climate pandemic”?
- Why not allow (postgraduate) medical training, national and international team meetings, scientific conferences [15] and networking events to benefit from this long-established technology?

The next premiere is just around the corner - the curtain will rise soon. So soon in fact that even civil disobedience of health professionals is being discussed in high-impact journals [16]. Therefore, we really need to reflect, focus, and revise – while there is time.

## Competing interests

The authors declare that they have no competing interests. 

## References

[1] Li HOY, Bailey AM (2020). Medical Education Amid the COVID-19 Pandemic: New Perspectives for the Future. Acad Med.

[2] Wang JJ, Deng A, Tsui BC (2020). COVID-19: novel pandemic, novel generation of medical students. Br J Anaesthesia.

[3] Aron JA, Bulteel AJ, Clayman KA, Cornett JA, Filtz K, Heneghan L, Hubbell KT, Huff R, Richter AJ, Yu K, Weil HF (2020). A Role for Telemedicine in Medical Education During the COVID-19 Pandemic. Acad Med.

[4] Abbasi S, Ayoob T, Malik A, Memon SI (2020). Perceptions of students regarding E-learning during Covid-19 at a private medical college. Pak J Med Sci.

[5] Chao TN, Frost AS, Brody RM, Byrnes YM, Cannady SB, Luu NN, Rajasekaran K, Shanti RM, Silberthau KR, Triantafillou Y, Newman JG (2020). Creation of an Interactive Virtual Surgical Rotation for Undergraduate Medical Education During the COVID-19 Pandemic. J Surg Educ.

[6] Chandratre S (2020). Medical Students and COVID-19: Challenges and Supportive Strategies. J Med Educ Curr Develop.

[7] Rosa H (2016). Resonanz: Eine Soziologie der Weltbeziehung.

[8] Nikendei C, Bugaj TJ, Nikendei F, Kühl SMK (2020). Klimawandel: Ursachen, Folgen, Lösungsansätze und Implikationen für das Gesundheitswesen. Z Evid Fortbild Qual Gesundhswes.

[9] Hagedorn G, Kalmus P, Mann M, Vicca S, Van den Berge J, van Ypersele JP, Bourg D, Rotmans J, Kaaronen R, Rahmstorf S, Kromp-Kolb H, Kirchengast G, Knutti R, Seneviratne SI, Thalmann P, Cretney R, Green A, Anderson K, Hedberg M, Nilsson D, Kuttner A, Hayhoe K (2019). Concerns of young protesters are justified. Science.

[10] Watts N, Amann M, Arnell N, Ayeb-Karlsson S, Belesova K, Boykoff M (2019). The 2019 report of The Lancet Countdown on health and climate change: ensuring that the health of a child born today is not defined by a changing climate. Lancet.

[11] Nikendei C (2020). After the game is before the game!. GMS J Med Educ.

[12] Bugaj TJ, Cranz A, Nikendei C (2020). The health-care sector's role in climate stabilisation. Lancet.

[13] Nikendei C, Cranz A, Bugaj TJ (2020). Two slides to make you think: 2slides4future, an initiative for teachers and lecturers advocating climate change education and teacher-learner dialogue. Med Educ.

[14] Litke N, Szecsenyi J, Wensing M, Weis A (2020). Green Hospitals: Klimaschutz im Krankenhaus. Dtsch Arztebl Int.

[15] Wynes S, Donner SD, Tannason S, Nabors N (2019). Academic air travel has a limited influence on professional success. J Clean Product.

[16] Bennett H, Macmillan A, Jones R, Blaiklock A, McMillan J (2020). Should health professionals participate in civil disobedience in response to the climate change health emergency?. Lancet.

